# *Coxiella burnetii* in Pakistan: a meta-analysis

**DOI:** 10.1186/s12917-025-05215-8

**Published:** 2025-12-18

**Authors:** Furqan Munir, Amna Shakoor, Hans-Peter Fuehrer, Muhammad Danial Wazir, Farakh Munir, Zia ud Din Sindhu

**Affiliations:** 1 Department of Parasitology, University of Agriculture, Faisalabad, Punjab 38040 Pakistan; 2 Institute of Parasitology, Centre of Pathobiology, Department of Biological Sciences and Pathobiology, University of Veterinary Medicine Vienna, Veterinärplatz 1, Vienna, 1210 Austria; 3 Key Laboratory of Animal Biotechnology of the Ministry of Agriculture of China, College of Veterinary Medicine, Northwest Agriculture and Forestry University, Yangling, Shaanxi Province 712100 China; 4 Department of Internal Medicine, Aziz Fatimah Medical and Dental College, Faisalabad, Punjab 38000 Pakistan; 5 Department of Research and Development, Air Pharmaceuticals, Kasur, Punjab 55050 Pakistan

**Keywords:** Q fever, *Coxiella burnetii*, Meta-analysis, Animals, Pakistan

## Abstract

*Coxiella* (*C.*) *burnetii* is a zoonotic pathogen that can infect humans, domestic and wildlife animals, and non-mammalian species. The pathogen is prevalent worldwide and exhibits high environmental stability; therefore, it has “One Health” significance. The objective of this meta-analysis was to summarize findings from studies about *C. burnetii* in Pakistan and included 19 published articles from 2015 to 2023. The collection and evaluation of scientific literature published on *C. burnetii* in Pakistan were done in accordance with PRISMA criteria. Three internet databases—Google Scholar, PubMed, and Science Direct—were used to find published research articles. The results showed 19 eligible studies involving small ruminants, large ruminants, camels, soil, ticks, and women from three provinces of Pakistan. The occurrence of infection was high in camels (31.3%) based on one study only, which limits its reliability, medium in goats and sheep (20.39% and 20.01% respectively), and low in women (8.41%). The maximum number of studies was conducted in Punjab (89.47%). The current study found that published information was scarce on the prevalence and distribution of *C. burnetii* in animals, humans (including men, women, and children), and the environment. The high heterogeneity was also observed in the estimated pooled prevalence. Future research might be focused on addressing these limitations and filling the gaps between the veterinary and human health systems. Improving diagnostics, standardizing reporting, and conducting large-scale studies are essential for estimating the real impact of Q fever and coxiellosis on public health and animals in Pakistan.

**Supplementary Information:**

The online version contains supplementary material available at 10.1186/s12917-025-05215-8.

## Introduction

*Coxiella burnetii* is a Gram-negative, intracellular, aerobic bacterium belonging to the family Coxiellaceae. It is the causative agent of Q fever in humans and coxiellosis in animals. It has been detected in various species such as rodents, birds, wildlife animals, and livestock, but the main reservoir hosts are sheep, goats, and cattle [1]. The pathogen is prevalent worldwide and exhibits environmental stability and a low infectious dose; therefore, it occurs as an emerging pathogen. Most human outbreaks occur because of their association with ruminants [2]. The primary route of transmission to humans is through inhalation of contaminated aerosols containing bacteria (the aerosol dose of infection is less than 10 bacterial cells) shed in the feces, milk, urine, and birth products of infected animals. The uncommon routes for the transmission of pathogens to humans are oral or blood transfusion. Individuals with occupational exposure, such as veterinarians and farmers, are at increased risk of getting infection [3]. Infected goats and cattle primarily shed bacteria in their milk, while sheep tend to shed large quantities of bacteria in the vaginal secretions. It is evidenced that bacteria are viable to some extent in the unpasteurized milk of infected animals, which has the potential of spreading to healthy individuals by consuming unpasteurized milk [4, 5].

Q fever first time was diagnosed in abattoir workers in Australia in 1935, and after two years, the pathogen was isolated from ticks. There is a debate about the role of ticks in transmitting *C. burnetii,* and some *Coxiella*-like species are endosymbionts of ticks necessary for the normal functioning of the ticks [6], and these endosymbiotic *Coxiella*-like species are non-pathogenic for vertebrates [7]. *C. burnetii* was isolated from various species of ticks that become infected transovarially or through ingestion of blood meal from infected animals [8]. Until the late 1990 s, the screening of ticks for the presence of *C. burnetii* was based on staining, morphological observations, and immunodetection techniques because the bacterium was difficult to culture [9]. However, advancement in the development of molecular biology showed that ticks harbour *Coxiella*-like endosymbionts, which are genetically distinct from *C. burnetii,* and the epidemiology of Q fever via ticks remains controversial. The ability of ticks to vector *C. burnetii* under field conditions seems limited and may occasionally transmit the bacterium to vertebrates, which might also be the secondary route for the transmission of the disease, as compared to the airborne transmission of the disease (reviewed in [10, 11]).

Most outbreaks of Q fever reported till now were mainly due to infected ruminants, indicating the importance of early detection and control of *C. burnetii* in these animals for the prevention of pathogen transmission to humans [12]. Clinically, Q fever in humans is characterized by fever, atypical pneumonia, hepatitis, neurological anomalies, and endocarditis. In animals, coxiellosis is often asymptomatic, but it can cause abortion storms, which are typically the primary clinical sign. Although the infection is often mild but it can lead to reproductive disorders such as endometritis, stillbirth, and infertility. Additionally, the shedding of bacteria also occurs during parturition or abortion storms, which pose a disease transmission risk to humans [12]. *C. burnetii* is highly resilient and capable of surviving for a longer time in soil and retaining infectivity under dry conditions. Wind or certain activities, such as construction, farming, and grazing of animals, can aerosolize the bacterial particles that pose a considerable inhalation risk. Therefore, sampling of the soil for the detection of environmental reservoirs for the bacteria and their transmission pathways is important [13]. The disease can be diagnosed with ELISA, indirect immunofluorescence assay (IFA), and polymerase chain reaction (PCR). Specific antibodies such as anti-IgM, IgG, and IgA can be used for both IFA and ELISA. Certain genes of *C. burnetii* can be amplified in PCR for the diagnosis of the disease or bacterium, e.g., IS1111 transposase gene, isocitrate dehydrogenase (icd) genes, com1 gene, and QpH1 or QpRS [14].

The disease can be effectively controlled through measures such as pasteurization of milk, culling of infected animals, and disinfecting contaminated areas. Additionally, antibiotics can be used for the treatment of diseased animals or infected humans, and vaccination of animals, as a prophylaxis measure can be performed [15]. This meta-analysis aims to provide updated scientific evidence on the prevalence of *Coxiella* infection in animals, humans, and the environment in Pakistan. This study further highlights the risk of infections in domestic animals in Pakistan.

## Materials and methods

This study was conducted according to the guidelines of Preferred Reporting Items for Systematic Reviews and Meta-analysis (PRISMA) [16]. The steps followed were the search for sensible literature, predefined standards for the inclusion and exclusion of research papers, and finding the relevant information to fulfill the objectives of this meta-analysis.

### Literature search

A search of the literature was conducted to find out the studies published from 1947 to August 2023 on *C. burnetii* in Pakistan using three databases, i.e., Google Scholar, PubMed, and Science Direct. The keywords used for the search include "*Coxiella burnetii*" or "*Coxiella*" and "*Coxiella* infection in Pakistan" or "*Coxiella burnetii* infection in Pakistan” and "Q fever" or "Q fever in Pakistan" and "Prevalence of *Coxiella burnetii* in Pakistan" or "Prevalence of *Coxiella* in Pakistan" and "Prevalence of Q fever in Pakistan" and "Epidemiology of Q fever in Pakistan" or "Epidemiology of *Coxiella* in Pakistan". Keywords were also used in combinations to fetch research articles in Pakistan. The bibliography available at the end of each retrieved research paper was also evaluated to identify the relevant articles (accessed until 31 August 2023).

### Quality assessment and selection

The selection criteria and assessment of the literature are presented in Supplementary Table 1. After the collection of literature from different sources (online database), based on the title and abstract, an initial exploration was performed to remove the irrelevant and duplicate research papers. Full-text paid articles were retrieved via the MOE Joint International Research Laboratory of Animal Health and Food Safety, College of Veterinary Medicine, Nanjing Agriculture University, Nanjing 210095, China. Furthermore, a second step was also taken to exclude those papers that were unavailable as full text, and priority was given to published articles.

**Fig. 1:**
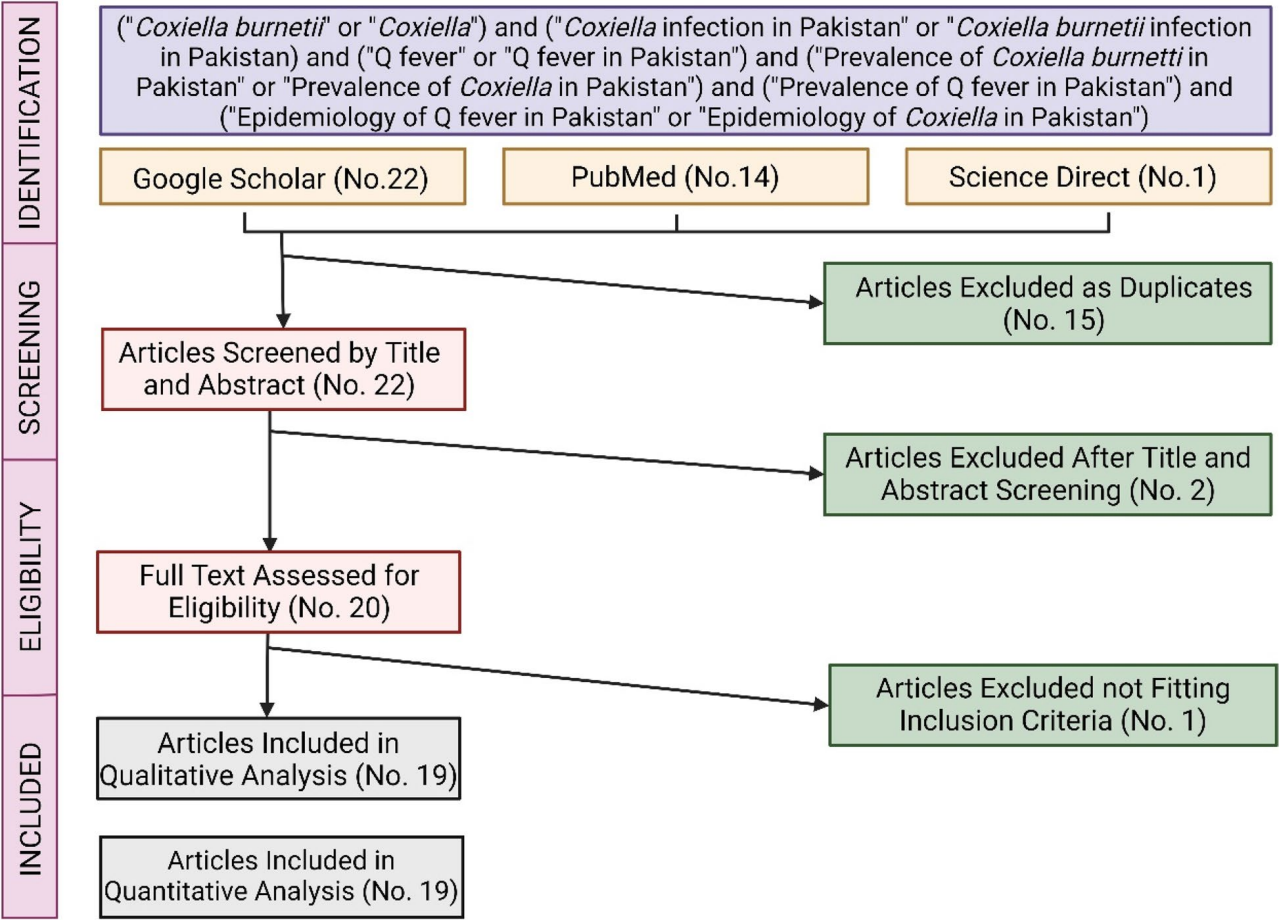
Flow diagram of the study selection process. The figure summarizes the number of research articles identified through database searching, the number of articles screened, full-text articles assessed for eligibility, and studies included in the final meta-analysis

A total pool of 37 entries (i.e., 22 from Google Scholar, 14 from PubMed, and 1 from Science Direct) was retrieved as shown in Fig. 1. Out of the total pool, 15 articles (40.54% of the original pool) were removed because they were identified as duplicate entries. A further 2 articles (5.40% of the original pool) were removed based on the screening by title and abstract. A total of 20 entries (54.05% of the original pool) by their full text were eventually evaluated and reviewed: out of them, 1 article (2.7% of the original pool) did not fit the inclusion criteria, therefore, was removed from the analysis. Only 19 (51.35% of the initial articles) fulfilled the inclusion criteria and were qualitatively and quantitatively analyzed. Eventually, information was extracted about the species, infection rate, location, study type and period, methodology, and reported prevalence.

### Statistical analysis

Initially, all the estimates from the included studies for animal species were summarized through a descriptive analysis. In some studies where raw data were not given, the prevalent cases were reverse-estimated (it involves calculating or estimating unavailable data points, e.g., from the percentage prevalence reported in the articles) from the available data. Crude prevalence was estimated as per 100 animals. Bivariate analysis was applied to estimate the risk ratios for the positive cases of domestic animals by assuming the positive status of soil retrieved from the studies as a reference group.

Systematic error or bias is any process that can occur during a study at any stage, leading to variations of results from the real value systematically [17]. Therefore, the study having bias could invalidate and compromise the results. There are various tools for examining the bias in the study. Nineteen research articles were included in the present study and analyzed for quality to prevent any potential bias from the study. Self-made questionnaires and quality eligibility criteria were used to assess the articles according to the Joanna Briggs Institute (JBI) critical appraisal checklist (JBI Evidence Synthesis Manual) and Cochrane and Grade handbook guidelines for systematic review assessments [18]. The variables included in the risk assessment include publication year, location, sample types, host species, and methods. The scoring of studies was done based on the questions (Supplementary Table 2). These questions were: (i) Was the sampling size appropriate? (ii) Were the samples selected randomly? (iii) Were the titles and abstracts of research papers relevant to the current study design? (iv) Was the sampling done appropriately? (v) Were the methods and analysis of data clear? (vi) Were the interpretation of results, statistical analysis, and discussion taking into account the objective of the study? All the papers were evaluated based on these six questions and scored at 6/6 × 100. The papers having 70% or above scores were considered high-quality papers, between 50 to 69% average quality papers, and low-quality papers having scores equal to or below 49% Supplementary Table 3.

In epidemiology, prevalence is defined as the proportion of hosts infected or infested with at least one individual pathogen divided by the total number of hosts examined for that pathogen [19]. We collated the data on the prevalence of many aspects from the studies in different regions of the country, where possible, and then estimated the overall prevalence in Pakistan using cumulative population data in an Excel spreadsheet (Microsoft 365®).

Pooled Odds Ratios (OR), prevalence estimates, and their corresponding 95% confidence interval as a meta-analysis of retrieved studies were calculated. Over a fixed effect model, a random effect model was preferred to withstand the heterogeneity in the design of the study. The heterogeneity of the retrieved studies was quantified by employing the I^2^ Statistic. Its categories include low heterogeneity, I^2^ ranging from 0 to 25%, moderate heterogeneity, I^2^ ranging from 26 to 50%, and substantial heterogeneity, I^2^ greater than 50%. To figure out possible reasons for high heterogeneity, a subgroup analysis was also performed by applying both the common effect model and the random effect model. Subgroup analysis is used to estimate whether the effect of an intervention differs across specific subgroups of studies that share certain characteristics, such as species, region, or study design. It helps to identify the potential variation or heterogeneity within different groups [17].

Funnel plots were plotted to ascertain the potential publication bias, and Egger’s test was eventually applied to check their asymmetry. Their Radial plots were generated to assess the small study bias. All these calculations were performed in R (version 4.3.1) and RStudio (version 2023.06.0 Build 421; RStudio, PBC; Boston, USA) software using the packages fmsb (version 0.7.5) and meta (version 6.5–0).

## Results

### Descriptive analysis

**Fig. 2:**
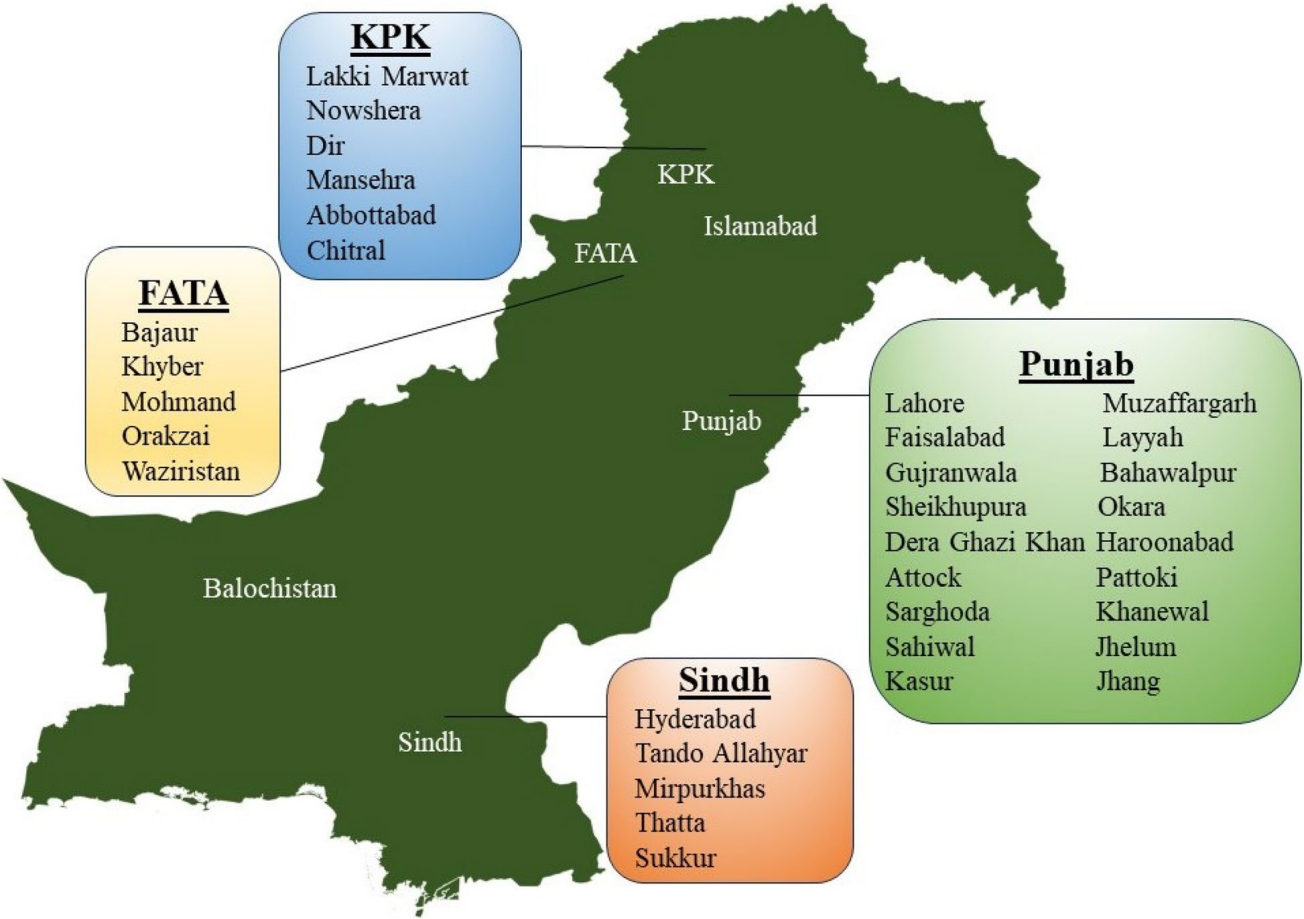
Geographic distribution of reported *Coxiella burnetii* infection in Pakistan. The map highlights regions where the prevalence of the pathogen was documented in the retrieved studies

The 19 research articles included in the meta-analysis, all of them published from 2015 to 2023, are summarized in Table 1 [20–38]. All the studies were based on polymerase chain reaction (PCR) and enzyme-linked immunosorbent assay, and included a total of 10,406 samples: 2,570 (24.69%) were soil samples, 7,539 samples (72.44%) were associated with animal species, and 297 samples (2.85%) were associated with humans. The majority of studies (17 out of 19, 89.47%) reported data from Punjab, a province of Pakistan having highest population including a total 9908 samples (95.21% from total samples in context of percentage, range 65 to 1055 samples per paper) [20–28, 28, 29, 29, 30, 30–32, 32, 33, 33, 34, 34, 35, 35, 36, 36, 37], followed by Sindh (2 studies out of 19, 10.52%) for a total 217 samples (2.08% from total samples, range 39 to 178 samples per paper) [26, 31]. Khyber Pakhtunkhwa (1 study, 5.26%) with a total of 227 samples (2.18% from total samples) [38] and FATA (1 study, 5.26%) with a total of 54 samples (0.51% from total samples) [27]. The regions covered by these studies are presented in Fig. 2.

**Table 1:** Summary of retrieved studies on *Coxiella burnetii* prevalence in different regions of Pakistan

Study references	Region	Time frame	Total number of samples	Source of sample	Number of examined samples	Number of positive samples (n; Percentage)
Shabbir et al. 2015 [20]	Punjab	2015^a^	145	Soil	145	7; 4.8%
Shabbir et al. 2016 [21]	Punjab	2011–2014	2425	Soil	2425	47; 1.94%
			464	Sheep	184	33; 17.9%
				Goat	280	46; 16.4%
Zahid et al. 2016 [22]	Punjab	2016	542	Sheep	271	77; 28.4%
				Goat	271	90; 33.2%
Ullah et al. 2019 [23]	Punjab	2016	1000	Sheep	500	78; 15.6%
				Goat	500	75; 15.0%
Rashid et al. 2019 [24]	Punjab	2018	827	Cattle	419	32; 7.6%
				Buffaloes	408	18; 4.4%
Ullah et al. 2019 [25]	Punjab	2019	1055	Sheep	500	78; 15.6%
				Goat	500	75; 15%
				Ticks	29	9; 31%
				Ticks	26	2; 26%
Ghafar et al. 2020 [26]	Punjab and Sindh	September to November, 2017	234	Ticks	234	3; 1.3%
Ghafar et al. 2020 [27]	FATA	2020	54	Ticks	54	4; 7.4%
Hussain et al. 2021 [28]	Punjab	2021	65	*Hyalomma* (Ticks)	45	18; 40%
				*Rhipicephalus* (Ticks)	20	8; 40%
Iqbal et al. 2021 [29]	Punjab	2020	320	Cattle	160	38; 32.12%
				Buffaloes	160	15; 12.5%
Iqbal et al. 2021 [30]	Punjab	2020	320	Sheep	160	75; 46.9%
				Goat	160	48; 30%
Memon et al. 2022 [31]	Sindh	2020	178	Sheep	32	12; 37.50%
				Goat	146	66; 45.20%
Ali et al. 2022 [32]	Punjab	2021	297	Human (Women)	297	25; 8.4%
Amin et al. 2022 [33]	Punjab	2021	300	Sheep	142	7; 4.9%
				Goat	158	27; 17.1%
Hussain et al. 2022 [34]	Punjab	2021	920	Dromedary camel	920	288; 31.30%
Hussain et al. 2022 [35]	Punjab	October 2020 to January 2021	448	Cattle	224	53; 23.66%
				Buffaloes	224	61; 27.23%
Zeeshan et al. 2023 [36]	Punjab	2022	385	Sheep	150	19; 12.6%
				Goat	235	31; 13.1%
Shujat et al. 2023 [37]	Punjab	2021–2022	200	Sheep (Meat)	50	19; 38%
				Goat (Meat)	50	11; 22%
				Cattle (Meat)	50	6; 12%
				Buffaloes (Meat)	50	4; 8%
Ali et al. 2023 [38]	Khyber Pakhtunkhwa	September 2021 to August 2022	227	Ticks	227	15; 6.7%

n: number

^a^For research papers in which the study period was not mentioned, the study period was estimated from the article's submission date

Total estimates of studies on the domestic animal species as well as soil included: 2 studies on the soil for total of 2570 samples (24.69%, range of samples 145 to 2425) [20, 21], 9 studies on the sheep for total 1989 samples (19.11%, range 50 to 500) [21–23, 25, 30, 31, 33, 36, 37], 9 studies on the goat for a total 2300 samples (22%, range 50 to 500) [21–23, 25, 30, 31, 33, 36, 37], 4 studies on the cattle for a total 853 samples (8.19%, range 50 to 419) [24, 29, 35, 37], 4 studies on the buffaloes for a total 842 samples (8.09%, range 50 to 408) [24, 29, 35, 37], 1 study on the dromedary camel for a total 920 samples (8.84%) [34], 1 estimate on the humans (women) for a total 297 samples (2.85%) and no studies were conducted on men and children [32], and 5 estimates on the ticks for a total 635 samples (6.1%, range 20 to 234) [25–28, 38].

As shown in Table 2, the crude prevalence for *Coxiella* was highest among dromedary camels (31.3%); however, this was based on only one study conducted on camels, which limits its reliability [34]. Goats were ranked second for crude prevalence (20.39%) and sheep at third position for crude prevalence (20.01%) [21–23, 25, 30, 31, 33, 36, 37]. The prevalence in the other species and soil in decreasing order were cattle (15.12%), buffaloes (11.63%), ticks (9.29%), humans (8.41%), and soil (2.1%) [20, 21, 24, 25, 28, 29, 32, 35, 37, 38].

**Table 2:** Crude estimates of *Coxiella burnetii* in animals and soil

	**Total** **(N/10,406; Percentage)**	**Positive** **(n/1,520; Percentage)**	**Prevalence**	**Risk ratio**	**95% CI (Lower limit-Upper limit)**
Soil	2570; 24.69%	54; 3.55%	2.10%	1	REFERENCE
Sheep	1989; 19.11%	398; 26.18%	20.01%	9.25	7.12% to 12.73%
Goats	2300; 22%	469; 30.85%	20.39%	9.7	7.28% to 12.93%
Cattle	853; 8.19%	129; 8.48%	15.12%	7.2	5.19% to 9.98%
Buffaloes	842; 8.09%	98; 6.44%	11.63%	5.54	3.94% to 7.79%
Camels	920; 8.84%	288; 18.94%	31.30%	14.9	11.03% to 20.12%
Humans	297; 2.85%	25; 1.64%	8.41%	4.01	2.46% to 6.53%
Ticks	635; 6.10%	59; 3.88%	9.29%	4.42	3.03% to 6.46%

N: number of samples examined; n: number of samples positive for *Coxiella burnetii*, *CI* confidence interval

### Risk of bias

**Fig. 3:**
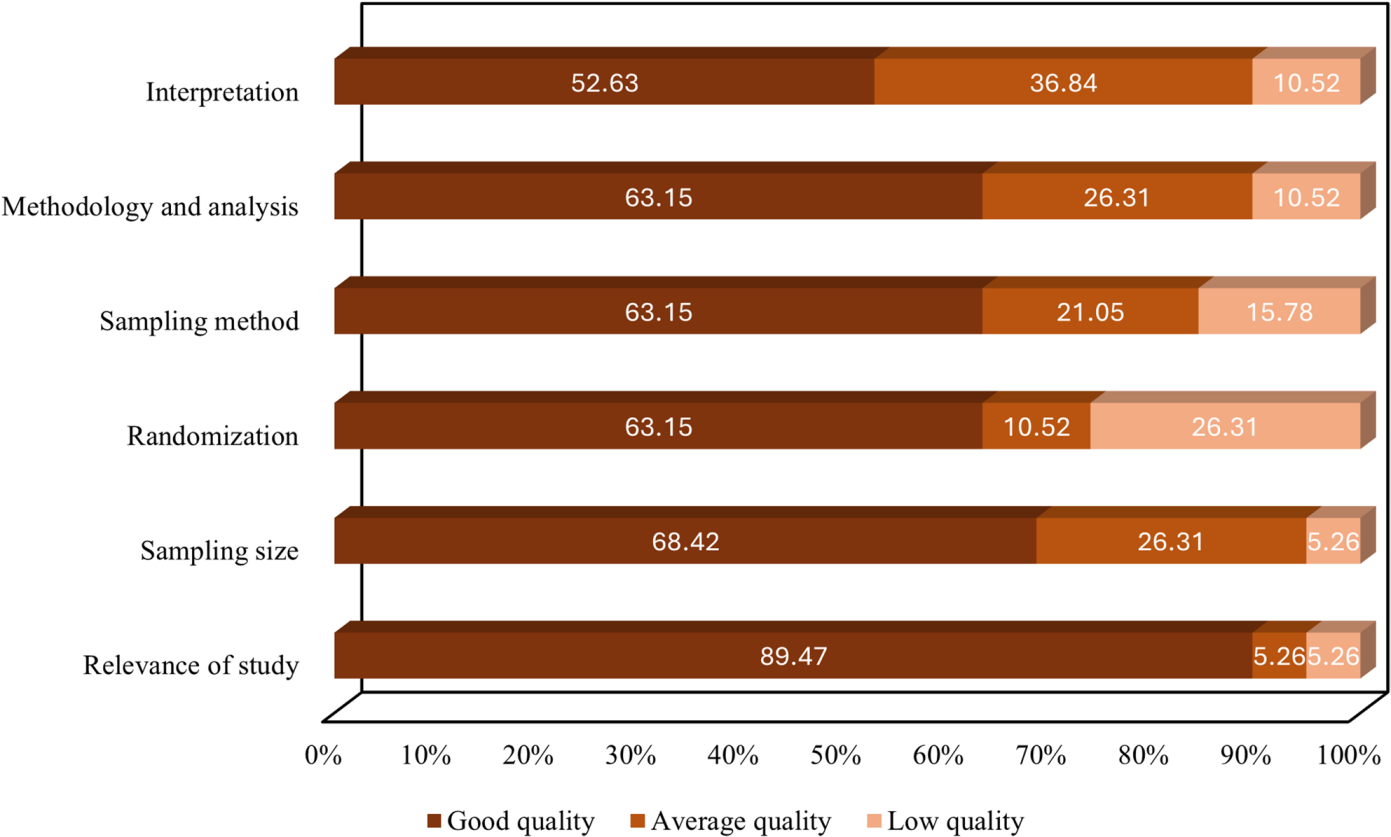
Proportional (%) risk of bias assessment of the retrieved studies. The figure represents the percentage of studies classified as good quality, average quality, or low quality based on the risk of bias evaluation criteria

A summary of the risk of bias in the retrieved studies is indicated in Fig. 3. The majority of the studies were of good quality. Only a few studies fell under 50% and were of low quality.

### Meta-analysis

Pooled prevalence rates for *Coxiella burnetii* in animals were estimated through a random model meta-analysis, and estimates for individual groups are indicated in Table 3.

**Table 3:** Results of a random-effect model meta-analysis on different species of animals for the prevalence status of *Coxiella burnetii* in the assessed groups

	**Prevalence per 100 animals**	**95% CI (Lower limit-Upper limit)**	**τ**^**2**^	**I**^**2**^	**Q**	**p**
Sheep	20.01%	18.27% to 21.83%	0.0177	94.20%	137.31	< 0.0001
Goats	20.39%	18.76% to 22.09%	0.0106	91.60%	95.39	< 0.0001
Cattle	15.12%	12.78% to 17.70%	0.0063	92.60%	40.35	< 0.0001
Buffaloes	11.63%	9.55% to 13.99%	0.0097	94.40%	53.82	< 0.0001
Dromedary camels	31.30%	28.31% to 34.41%	-^a^	-	-	-
Humans	8.41%	5.52% to 12.17%	-	-	-	-
Ticks	9.29%	7.14% to 11.82%	0.0207	93.20%	58.78	< 0.0001

*CI* Confidence Interval

^a^A random-effect model meta-analysis is not possible when the sample size is only one

Estimates were 20.01% (95% CI: 18.27 to 21.83) among sheep, 20.39% (95% CI: 18.76 to 22.09) among goats, 15.12% (95% CI: 12.78 to 17.7) among cattle, 11.63% (95% CI: 9.55 to 13.99) among buffaloes, 31.30% (95% CI: 28.31 to 34.41) among dromedary camels, 8.41% (95% CI: 5.52 to 12.17) among women, and 9.29% (95% CI: 7.14 to 11.82) among ticks. Most of the estimates were affected by substantial heterogeneity and more precisely sheep (I^2^ = 94.20%, Q = 137.31, *p* < 0.0001), goat (I^2^ = 91.60%, Q = 95.39, *p* < 0.0001), cattle (I^2^ = 92.6%, Q = 40.35, *p* < 0.0001), buffaloes (I^2^ = 94.40%, Q = 53.82, *p* < 0.0001), and ticks (I^2^ = 93.20%, Q = 58.78, *p* < 0.0001).

**Fig. 4:**
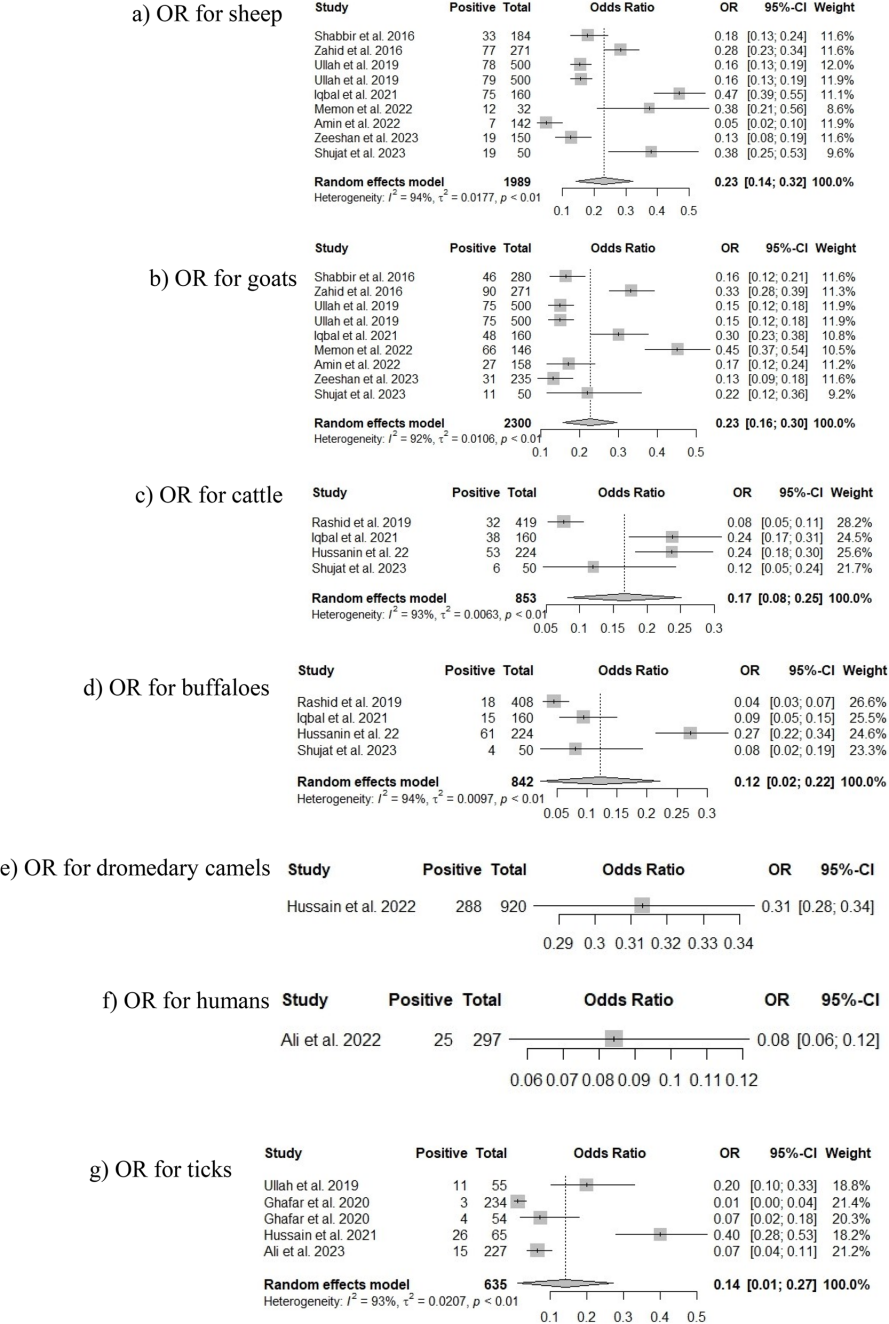
Forest plots representing the odds ratio (OR) of *Coxiella burnetii* infection across different host species. Subfigures show results for: **a** sheep, **b** goats, **c** cattle, **d** buffaloes, **e** dromedary camels, **f** humans, and **g** ticks. Each square represents the prevalence reported by a single study, and a horizontal line through the square shows the 95%-CI, while the diamond shape represents the pooled estimates or overall prevalence for a group of studies, and its width represents the 95%-CI

The prevalence rates for animal species and pooled odds ratios with corresponding 95% CI are provided in Fig. 4 (A1 to A7). Sheep were characterized by higher likelihood for *Coxiella burnetii* positive status (Fig. 3a; OR 0.23, 95% CI: 0.14 to 0.32), followed by goats (Fig. 3b; OR 0.23, 95% CI: 0.16 to 0.30), cattle (Fig. 3c; OR 0.17, 95% CI: 0.08 to 0.25), ticks (Fig. 3g; OR 0.14, 95% CI: 0.01 to 0.27), and buffaloes (Fig. 3d; OR 0.12, 95% CI: 0.02 to 0.22).

### Analysis of publication bias and small study bias

**Fig. 5:**
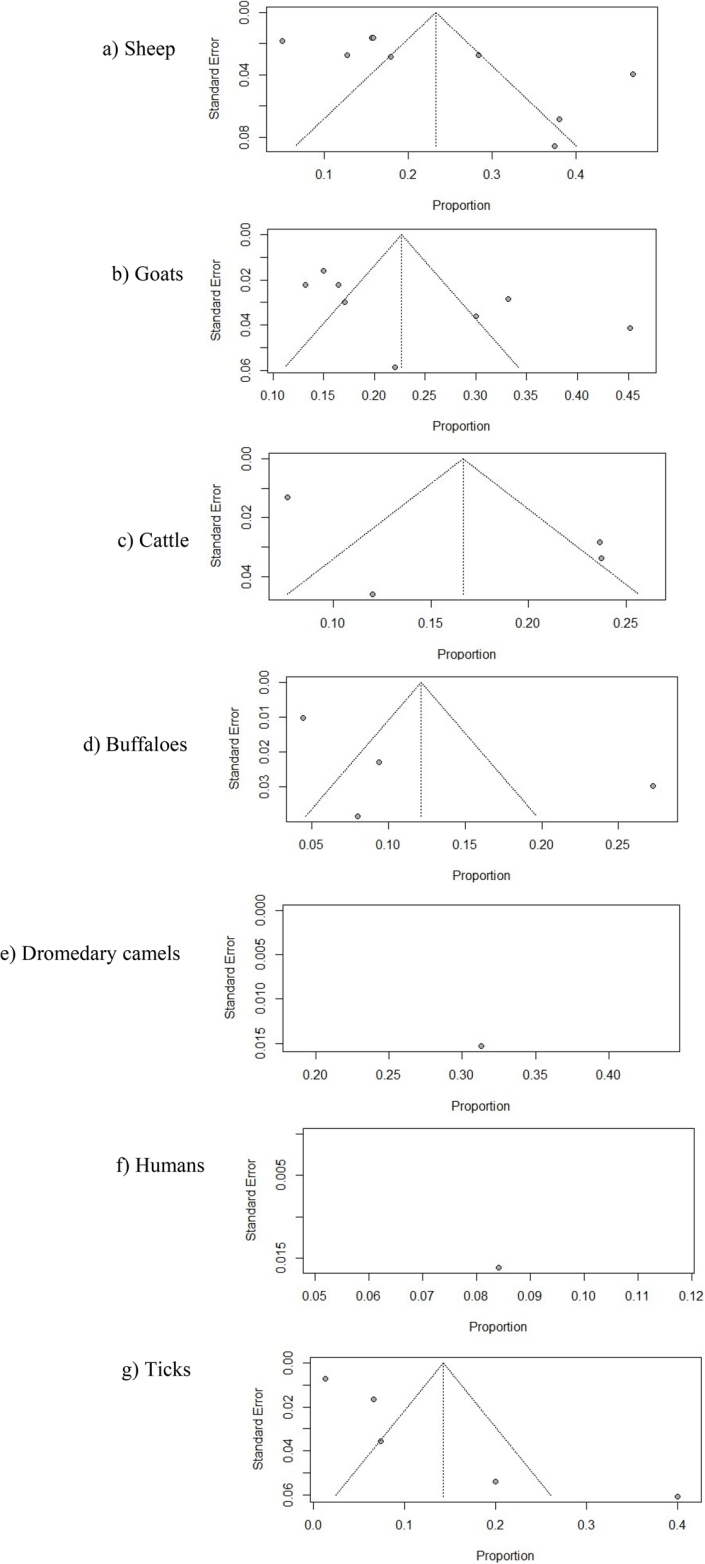
Funnel plots for assessing publication bias in the meta-analysis of *Coxiella burnetii* prevalence across different host species. Subfigures: **a** sheep; **b** goats; **c** cattle; **d** buffaloes; **e** dromedary camels; **f** humans; **g** ticks

**Fig. 6:**
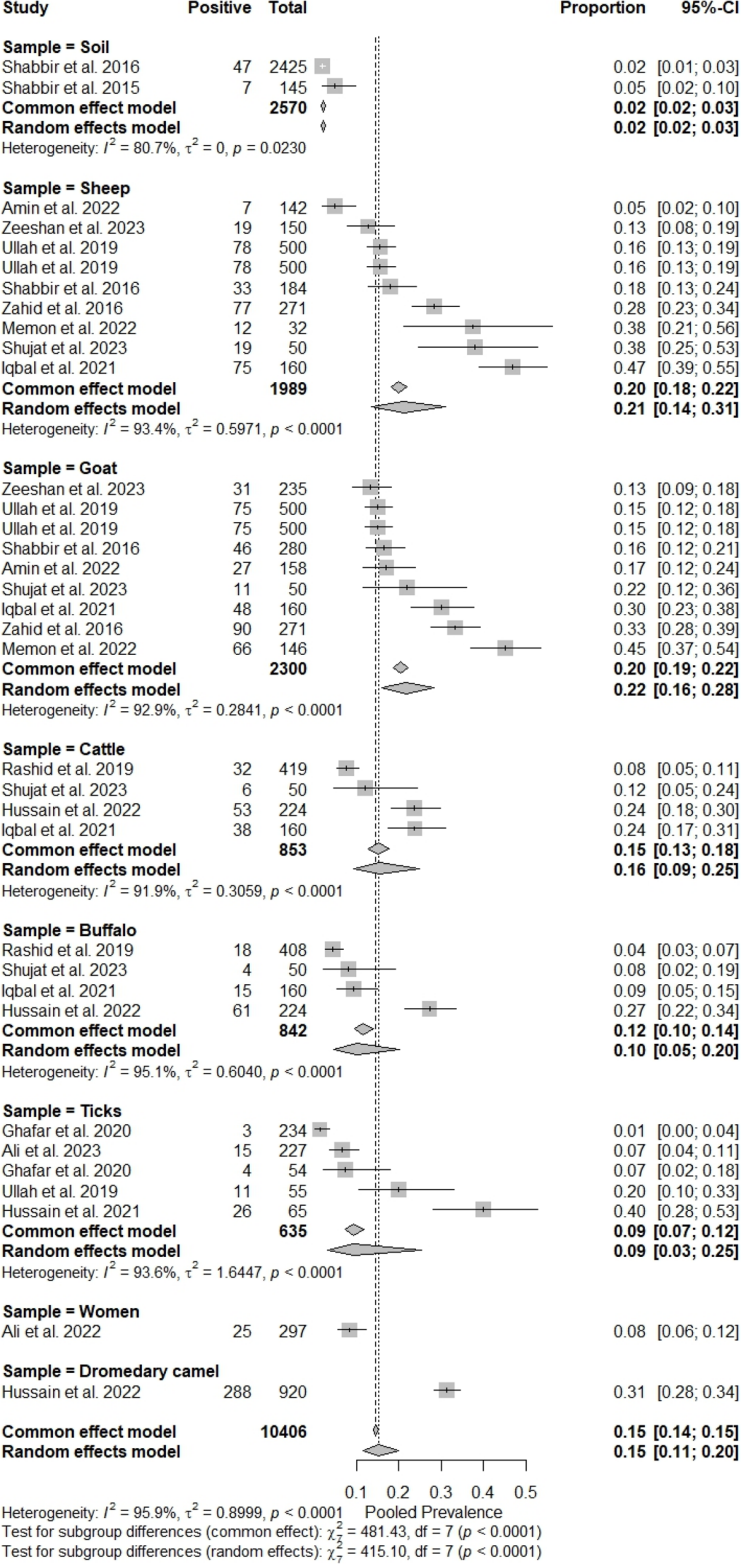
Subgroup analysis for the assessment of bias in the retrieved studies. Each square represents the prevalence reported by a single study, and a horizontal line through the square shows the 95%-CI, while the diamond shape represents the pooled estimates or overall prevalence for a group of studies, and its width represents the 95%-CI

Potential publication bias was evaluated in Fig. 5 by analysis of funnel plots. The graphical representation of an index of precision (Y-axis) or the sample size plotted against the effect size (X-axis) is known as a funnel plot [39]. Estimates converge around the true underlying effect size when the sample size increases. Therefore, when the analysis is not influenced by publication bias, the point estimates will be evenly scattered. On the other hand, the point estimates will be asymmetrically scattered when there is a publication bias, with fewer studies showing negative results than positive results. The visual inspection of Fig. 4 suggested that the point estimates are quite uneven, particularly for sheep, goats, cattle, buffaloes, and ticks. Egger’s test was applied to further confirm the publication bias, which is a linear regression analysis. In this analysis, the estimated intervention effects on their standard errors, weighed by their inverse variance, are evaluated. As shown in Table 4, analyses on sheep, goats, cattle, buffaloes, and ticks were characterized by substantial publication bias (*p* = 0.9786, *p* = 0.8844, *p* = 0.7369, *p* = 0.3384, and *p* = 0.2788, respectively). The subgroup analysis evaluating the effect of different sample groups on the substantial heterogeneity among studies is presented in Fig. 6. The pooled odds ratios obtained from the overall meta-analysis were slightly similar to those observed in the subgroup analysis. This indicates that the effect estimates were consistent across the examined subgroups and there was a non-significant variation in the association between subgroups. Therefore, the results obtained were uniform across different study groups, supporting the reliability or stability of the overall pooled estimates.

**Table 4:** Summary of Egger’s test for funnel plot asymmetry (SE = standard error)

	**t**	**df**	**Bias (SE)**	**Intercept (SE)**	***p*-value**
Sheep	0.03	7	3.7423	0.6515	0.9786
Goats	0.15	7	5.2579	0.8387	0.8844
Cattle	−0.39	2	6.9230	1.3560	0.7369
Buffaloes	−1.25	2	5.4558	1.2287	0.3384
Dromedary camels	-	-	-	-	-
Humans	-	-	-	-	-
Ticks	−1.32	3	5.3644	1.7867	0.2788

**Fig. 7:**
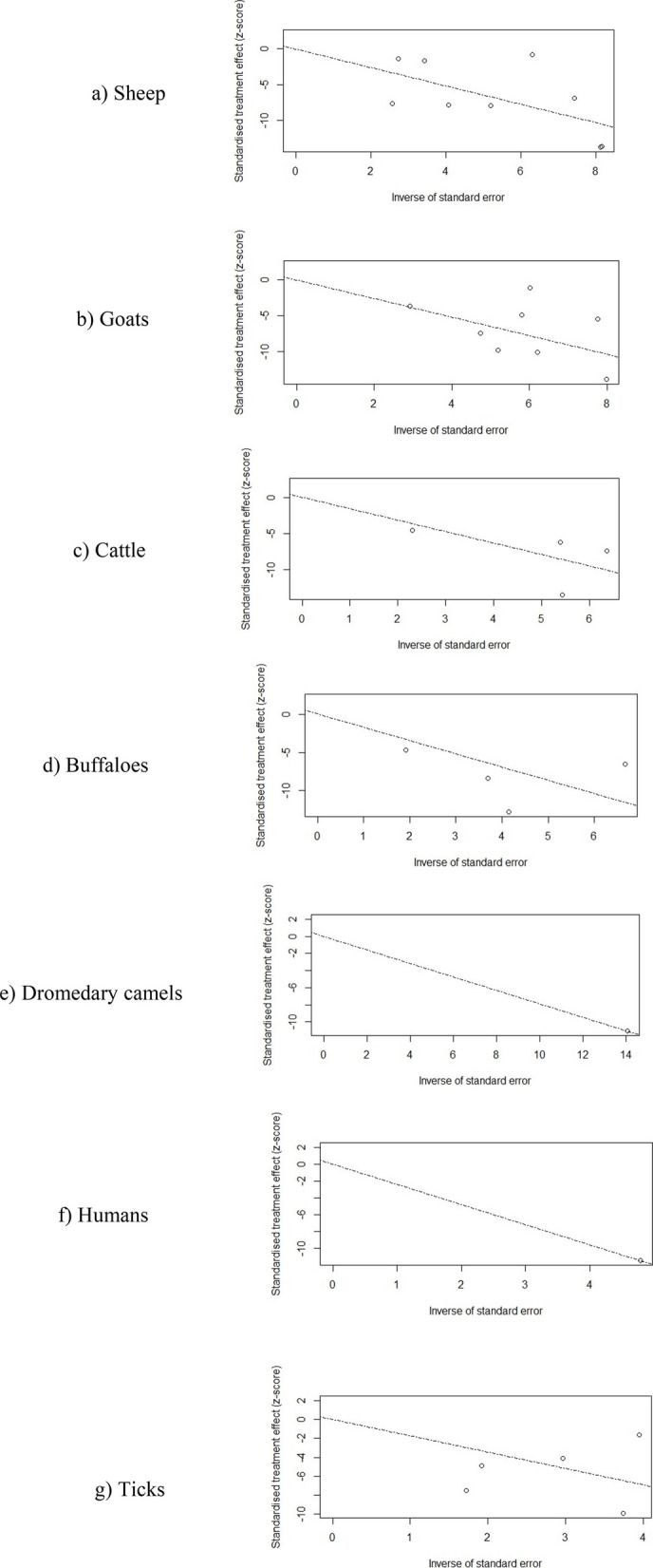
Radial plots for studies included in the meta-analysis for assessing small study bias across different host species. Subfigures: **a** sheep; **b** goats; **c** cattle; **d** buffaloes; **e** dromedary camels; **f** humans; **g** ticks

To examine the small study bias, radial plots have been calculated, and their corresponding graphs are provided in Fig. 7. To calculate the radial plots, first, z-statistics or standard estimates are calculated. It can be done by dividing each estimate by its standard error (SE). Their scatter plots can be prepared by plotting each z-statistic (Y-axis) against 1/SE (X-axis). Moreover, the larger studies have larger 1/SE and smaller SE, which will be observed away from the origin line. Small study bias can be evaluated by even plotting point estimates of studies alongside the regression line. Based on the visual inspection, small study bias can be ruled out reasonably among sheep, goats, cattle, buffaloes, and ticks.

## Discussion

*Coxiella burnetii* is stable in the environment, highly infective, and has an aerosol route of transmission. Thus, it is a potential risk not only for animals but also for humans [40]. Therefore, the data included in this meta-analysis, retrieved from different studies, indicated that it is prevalent at various locations in Pakistan and may act as a biological threat to the hosts. However, only a few studies have been conducted on Q fever or coxiellosis in Pakistan. The first research conducted on *Coxiella* in small ruminants and soil was from 2011 to 2014, and the results were published in 2016 by Shabbir et al. (2016) [21]. Before this period, the less attention of the researchers toward this pathogen may have been due to the asymptomatic occurrence of the disease and the unavailability of molecular and serological facilities, such as PCR or ELISA, in Pakistan. However, in recent years, from 2015 to 2023, an increase in studies on the prevalence of *Coxiella* infection in animals as well as soil was observed. This increase is due to an increase in the number of research institutes and universities in Pakistan equipped with high-standard laboratories. The facility of ELISA for the detection of antibodies against *Coxiella* infection and PCR has a significant contribution to the detection of infection in animals [41]. The serological assay is suitable for conducting large epidemiological surveys, but it can not detect pathogens in ticks. For detecting pathogens from ticks, PCR could be the best choice.

There are no existing published studies on *C. burnetii* infection from Balochistan. It is the largest province of Pakistan in terms of land, with less population when compared to other provinces. The people living there mostly adopt ethnoveterinary practices rather than advanced diagnostic tools. These could be the reasons for no work on Coxiellosis in Balochistan. Likewise, no existing published studies on *C. burnetii* infections in wild animals, pets, and birds are available. In Pakistan, resource-deficient people are dependent on donkeys and horses to earn their livelihood. Their health is essential for the farmers, as these animals are utilized for craft purposes, so that they can support their livelihood. No epidemiological study was conducted in equine related to coxiellosis. Additionally, only one study has been conducted in humans that was also focused on women only [32]. No information related to the prevalence of infection in men and children in Pakistan is available, which indicates a limitation for public health relevance. Similarly, a single study was conducted in camels, which indicated the high prevalence of *C. burnetii* infection in camelids in Pakistan [34]. Moreover, attention must be paid to finding the estimates of infection and its preventive strategies.

Ticks are a potential source for the transmission of various pathogens in animals. *C. burnetii* was also isolated from more than 40 different species of hard ticks; however, transmission of *C. burnetii* from ticks to animals in the field is not known. Only one study, conducted by Hussain et al. (2021) that demonstrates the presence of *C. burnetii* in various species of ticks in Punjab, Pakistan [28]. As this is a zoonotic pathogen, humans can acquire infection from infected animals when they inhale pathogens in the air shed by these animals or by consuming the contaminated animal products [13]. Additionally, researchers pay more attention to domestic animals because of their economic significance and the consumption of their products by people [4]. Farmers and abattoir workers handling these animals are at increased risk of infection. However, no such data is available that indicates the prevalence of infection in workers such as abattoir workers or milkmen.

A substantial heterogeneity was observed among the included studies, suggesting the variability in the estimated pooled prevalence, and multiple tests were performed, e.g., subgroup analysis to identify the source of this heterogeneity. However, none of the performed analyses accounted for the observed variability. The high heterogeneity may reflect the differences in estimating the correct sampling size, appropriate sample collection and methodology, lack of standardized reporting of prevalence, varying quality, limited control of confounding factors, and/or few or even one study on some species [17]. Finally, the epidemiology of Q fever in Pakistan remains overlooked and under-researched. As Pakistan is an agricultural country and its large population is associated with livestock [42]. Therefore, more research might be needed to estimate the prevalence of disease in animals and their products, humans, including men, women, and children, and the environment. A comprehensive One Health approach might be adopted to fill the gap between the veterinary and human health systems [43]. Improvement in the diagnostic tools, reporting standards, and well-designed, large-scale studies may provide the real impact of Q fever and coxiellosis on public health and animals in Pakistan.

## Conclusion

This study highlights the limited number of published studies on *Coxiella burnetii* in Pakistan, with most studies focusing primarily on domestic ruminants and ticks from domesticated animals. Only one study has investigated the infection in women, and there is a lack of research on men and children. These gaps indicate the need for comprehensive epidemiological studies across the country on domestic, wild, and pet animals, as well as humans. Such epidemiological studies would be helpful in determining the impact of pathogens on animals and public health, identifying the potential reservoirs, and assessing the associated risk factors.

## Supplementary Information

Supplementary Material 1.

## Data Availability

The data presented in this study are available within the article.

## Abbreviations

*Coxiella* (*C.*) *burnetii*: *Coxiella*
PRISMA: Preferred Reporting Items for Systematic Reviews and Meta-analysis
IFA: Immunofluorescence assay
ELISA: Enzyme-linked immunosorbent assay
iELISA: Indirect ELISA
PCR: Polymerase chain reaction
RT-PCR: Real Time-PCR
IgA: Immunoglobulin-A
IgG: Immunoglobulin-G
IgM: Immunoglobulin-M
ICD: Isocitrate dehydrogenase
Com1: *Coxiella* outer membrane protein 1
QpH1: A specific type of plasmid
QpRS: A specific type of plasmid
JBI: Joanna Briggs Institute
OR: Odd Ratio
CI: Confidence interval
τ^2^: Symbol used for heterogeneity variance
I^2^: Symbol used for the proportion of heterogeneity
Q: Symbol used for Cochran’s Q statistic
p: Symbol used for p-value that measures the probability
SE: Standard error
