# Supplementary material for: Coxiella burnetii in Pakistan: a meta-analysis
Source: BMC Vet Res. 2025 Dec 18;22:58. doi: 10.1186/s12917-025-05215-8 (PMC12849355; doi:10.1186/s12917-025-05215-8)
Supplement: Supplementary file 1 — Supplementary Material 1. [file 12917_2025_5215_MOESM1_ESM.docx]

| **Supplementary Table 1:** Specific criteria for study inclusion and exclusion. |
| --- |
| **Inclusion criteria** |
| The study must focus on *Coxiella burnetii.* |
| The research must be carried out in Pakistan or specific regions within Pakistan. |
| The studies must report the prevalence or incidence of *Coxiella burnetii* infection in humans, animals, or the environment. |
| Only original research articles should be included. |
| **Exclusion criteria** |
| Reviews, editorials, opinions, or studies without primary data |
| Studies with unavailable full-text papers |

| **Supplementary Table 2:** Eligibility assessment questions used to screen studies for meta-analysis. | | |  |
| --- | --- | --- | --- |
| **Sr. no.** | **Questions** | **Score** | **Weightage** |
| 1 | Were the titles and abstracts of research papers relevant to the current study design? | 1/6*100 | 16.66% |
| 2 | Was the sampling size appropriate? | 1/6*100 | 16.66% |
| 3 | Was the sample selected randomly? | 1/6*100 | 16.66% |
| 4 | Was the sampling done appropriately? | 1/6*100 | 16.66% |
| 5 | Were the methods and analysis of data clear? | 1/6*100 | 16.66% |
| 6 | Was the interpretation of results, statistical analysis, and discussion taking into account the objective of the study? | 1/6*100 | 16.66% |

| **Supplementary Table 3:** Quality assessment and scoring of studies based on the six questions. | | | | | | | |
| --- | --- | --- | --- | --- | --- | --- | --- |
| **Study** | **Q1** | **Q2** | **Q3** | **Q4** | **Q5** | **Q6** | **Justifications** |
| Shabbir et al. 2015 [20] |  |  |  |  |  |  | The study design, methodology, statistical analysis, interpretations, results, and discussions were clear and appropriately addressed. |
| Shabbir et al. 2016 [21] |  |  |  |  |  |  | The study design, methodology, statistical analysis, interpretations, results, and discussions were clear and appropriately addressed. |
| Zahid et al. 2016 [22] |  |  |  |  |  |  | This study addresses questions clearly; however, it scored above average on the last question. |
| Ullah et al. 2019 [23] |  |  |  |  |  |  | This study might not clearly address randomization; however, the sample size was large. The sampling methods, analysis, interpretation of results, and discussion were average. |
| Rashid et al. 2019 [24] |  |  |  |  |  |  | This study might not clearly address randomization, but the sample size is large. The methods and data analysis were scored as average, and the analysis and interpretation of results could be presented in more detail. |
| Ullah et al. 2019 [25] |  |  |  |  |  |  | The study design, methodology, statistical analysis, interpretations, results, and discussions were clear and appropriately addressed; however, the sampling size of ticks was quite small. |
| Ghafar et al. 2020 [26] |  |  |  |  |  |  | This study somewhat addresses the first and fifth questions. The presentation of the study for the remaining questions was appropriate |
| Ghafar et al. 2020 [27] |  |  |  |  |  |  | This study lacks proper presentation of methodology, such as sample size estimation, randomization, and appropriate sampling. The title and abstract of the study were above borderline in terms of meeting the inclusion criteria |
| Hussain et al. 2021 [28] |  |  |  |  |  |  | This study might not clearly address randomization in sample collection, but it scored above average on the remaining questions. |
| Iqbal et al. 2021 [29] |  |  |  |  |  |  | This study addresses questions clearly; however, it scored above average on the last question. |
| Iqbal et al. 2021 [30] |  |  |  |  |  |  | The study design, methodology, statistical analysis, interpretations, results, and discussions were clear and appropriately addressed. |
| Memon et al. 2022 [31] |  |  |  |  |  |  | This study might lack clear information on randomization of sample collection and adequate presentation of methods, analysis, interpretation of results, and discussion. |
| Ali et al. 2022 [32] |  |  |  |  |  |  | The methodology was not clear, e.g., inappropriate estimation of sample size, whether samples were randomly collected or not, and whether they were collected from patients who visited hospitals for routine checkups or from women working in the field. The result interpretation might be elaborated, e.g., distinguishing between the acute and chronic stages of infection. |
| Amin et al. 2022 [33] |  |  |  |  |  |  | The study design, methodology, statistical analysis, interpretations, results, and discussions were clear and appropriately addressed. |
| Hussain et al. 2022 [34] |  |  |  |  |  |  | This study clearly addresses the first five questions, and the interpretation regarding the last question was above average. |
| Hussain et al. 2022 [35] |  |  |  |  |  |  | The study design, methodology, statistical analysis, interpretations, results, and discussions were clear and appropriately addressed. |
| Zeeshan et al. 2023 [36] |  |  |  |  |  |  | The study design, methodology, statistical analysis, interpretations, results, and discussions were clear and appropriately addressed. |
| Shujat et al. 2023 [37] |  |  |  |  |  |  | The methodology of this study might be unclear, e.g., random sample collection and sample preservation during transportation are not mentioned. A questionnaire survey regarding animal health status, breed, sex, or origin might provide valuable information if included. |
| Ali et al. 2023 [38] |  |  |  |  |  |  | The sample size was not appropriate. However, this study clearly addresses the remaining five questions. |

Full score;Above average score;  Average or below average score; Q1: Were the titles and abstracts of research papers relevant to the current study design?; Q2: Was the sampling size appropriate?; Q3: Was samples selected randomly?; Q4: Was the sampling done appropriately?; Q5: Were the methods and analysis of data clear?; Q6: Was the interpretation of results, statistical analysis, and discussion taking into account the objective of the study?
